# Complete response to fifth-line anti-PD-1 rechallenge in fumarate hydratase-mutated papillary renal cell carcinoma

**DOI:** 10.1038/s41698-024-00750-3

**Published:** 2024-11-04

**Authors:** Isabella Portugal, Maria A. Clavijo-Salomon

**Affiliations:** 1 Ciências Médicas, Faculdade de Medicina da Universidade de São Paulo (FMUSP), São Paulo, Brazil; 2 Centro de Investigação Translacional em Oncologia (CTO), Instituto do Câncer do Estado de São Paulo (ICESP), Faculdade de Medicina da Universidade de São Paulo (FMUSP), São Paulo, Brazil; 3 Departamento de Imunologia, Instituto de Ciências Biomédicas, Universidade de São Paulo, São Paulo, Brazil; 4 Present Address: Department of Medicine, Cambridge Health Alliance, Cambridge, MA USA; 5 Present Address: Laboratory of Integrative Cancer Immunology, Center for Cancer Research, National Cancer Institute, NIH, Bethesda, MD USA

**Keywords:** Renal cell carcinoma, Prognostic markers, Cancer immunotherapy

## Abstract

Fumarate hydratase (*FH*) mutated papillary renal cell carcinoma is an aggressive variant of kidney cancer that poorly responds to conventional targeted therapies and immunotherapy. Here, we present the 10-year follow-up of a heavily pre-treated patient with several lines of therapy, achieving a remarkable complete response to anti-PD-1 rechallenge. In addition, we highlight a common immune-related adverse event of anti-PD-1, eosinophilia, as a possible biomarker of response and using TCGA data analysis, provide proof-of-concept for tumor expression of the eosinophil-related gene *SIGLEC8*, as a promising powerful predictor of prognosis for papillary renal cell carcinoma patients.

## Introduction

Fumarate hydratase (*FH*) mutated papillary renal cell carcinoma (pRCC), formerly known as type 2^1^, is a rare and aggressive form of kidney cancer often linked with hereditary leiomyomatosis (HLRCC) syndrome^2^. This variant arises from inactivating mutations in the *FH* gene, leading to the accumulation of fumarate, which disrupts cellular metabolism and promotes tumorigenesis^2–4^. The *FH*-deficient pRCC generally has a poor prognosis, with most patients presenting at advanced stages and having shorter 5-year survival than other types of pRCC due to rapid progression and metastasis^5,6^. As the *FH* mutation triggers the upregulation of pro-angiogenic growth factors, vascular growth factor inhibitors (VEGFi; bevacizumab), tyrosine kinase inhibitors (TKI; sunitinib, sorafenib, pazopanib, axitinib, and cabozantinib), mammalian target of rapamycin inhibitors (mTORi; everolimus and temsirolimus) and endothelium growth inhibitors (EGFRi; erlotinib) have been used alone and more recently in combination with immune checkpoint inhibitors (ICI; ipilimumab, nivolumab, pembrolizumab, atezolimumab, and durvalumab)^7–9^.

*FH*-mutated pRCC cells preferably carry out aerobic glycolysis, decreasing glucose and increasing lactate, leading immune cells in the microenvironment to anergy and immunosuppression^10,11^. Glucose deprivation represses calcium signaling, IFN-γ production, cytotoxicity, and motility in T cells; nevertheless, ICI re-energizes anabolic metabolism and glycolysis in exhausted T cells^12^. The standard of care for *FH*-mutated pRCC is still controversial and remains ultimately the physician’s recommendation^13^. Based on the European Society for Medical Oncology (ESMO) guidelines, monotherapy TKI with cabozantinib (over sunitinib) and pembrolizumab are currently considered standard of care for first-line advanced pRCC^14,15^. Still, the combination of erlotinib and bevacizumab has shown moderate side effects and long-lasting partial responses, considered by others the standard of care for HLRCC / pRCC patients with the *FH*-mutated nuance^13,16–19^. Although new targeted therapies for pRCC molecular subtypes continue to be explored, *FH*-mutated pRCC patients are underrepresented or included in basket immunotherapy trials as all non-clear cell RCC (nccRCC)^9^. Only three clinical trials are currently active or recruiting *FH*-mutated pRCC patients worldwide (Table 1)^20^. To date, five successful and one partial response cases of *FH*-mutated pRCC treated with first-line ipilimumab plus nivolumab and one successful case of HLRCC *FH-*mutated pRCC treated with first-line pembrolizumab have been reported^21–24^. Recently, a multicenter retrospective analysis of 91 patients with *FH*-deficient RCC found the first-line ICI plus TKI combination to have a significantly higher objective response rate (ORR) compared to TKI monotherapy^25^. In contrast, we present a comprehensive 10-year follow-up of a heavily pre-treated FH-mutated pRCC patient, initially non-responder to nivolumab but who presented a complete response to nivolumab rechallenge after a year of axitinib. ICI rechallenge is uncommon and in the context of metastatic RCC (mRCC) has largely demonstrated negative discouraging results^26–28^. Here, we present the first case of a heavily pre-treated patient with advanced *FH*-mutated pRCC presenting an exceptionally complete and sustained response to nivolumab rechallenge.

**Table 1:** Current clinical trials for fumarate hydratase-deficient renal cell carcinoma

NCT number	Study title	Conditions	Interventions	Study design	Study status	Sponsor
NCT05877820	A Study to Evaluate Efficacy and Safety of Lenvatinib Combined with Tislelizumab in Patients With FHRCC	FHRCC	BIOLOGICAL: Tislelizumab |DRUG: Lenvatinib	Allocation: NA |Intervention Model: SINGLE_GROUP |Masking: NONE |Primary Purpose: TREATMENT	RECRUITING	RenJi Hospital
NCT04387500	Sintilimab Injection Combined with Inlyta in Fumarate Hydratase- Deficient Renal Cell Carcinoma	RCC|FH-Deficiency	DRUG: Sintilimab injection plus Inlyta treatment	Allocation: NA |Intervention Model: SINGLE_GROUP. |Masking: NONE. |Primary Purpose: TREATMENT	ACTIVE; NOT RECRUITING	West China Hospital
NCT04068831	Talazoparib and Avelumab in Participants With Metastatic Renal Cell Carcinoma	Metastatic RCC|FHRCC | Succinate Dehydrogenase Deficient RCC	DRUG: Talazoparib |DRUG: Avelumab	Allocation: NON_RANDOMIZED |Intervention Model: SINGLE_GROUP |Masking: NONE |Primary Purpose: TREATMENT	COMPLETED	Memorial Sloan Kettering Cancer Center
NCT03635892	A Study of Nivolumab In Combination With Cabozantinib in Patients With Non-Clear Cell Renal Cell Carcinoma	Advanced or Metastatic Non-clear Cell RCC | Unclassified RCC | pRCC| FHRCC|SDH-Deficient RCC |Collecting Duct RCC |Chromophobe RCC	DRUG: cabozantinib |DRUG: nivolumab	Allocation: NON_RANDOMIZED |Intervention Model: SINGLE_GROUP |Masking: NONE |Primary Purpose: TREATMENT	ACTIVE; NOT RECRUITING	Memorial Sloan Kettering Cancer Center
NCT04146831	Sintilimab in FH-deficient Renal Cell Carcinoma	RCC | FH-deficient | Sintilimab	DRUG: Sintilimab	Allocation: NA |Intervention Model: SINGLE_GROUP. |Masking: NONE |Primary Purpose: TREATMENT	UNKNOWN	West China Hospital
NCT02071862	Study of the Glutaminase Inhibitor CB-839 in Solid Tumors	Solid Tumors | TNBC | NSCLC | RCC | Mesothelioma | FH-Deficient Tumors| SDH-Deficient GIST | SDH-Deficient Non-GIST | Tumors Harboring IDH1 and IDH2 Mutations | Tumors Harboring cMyc Amplifications	DRUG: CB-839 DRUG: Pac-CB DRUG: CBE DRUG: CB-Erl DRUG: CBD DRUG: CB-Cabo	Allocation: NON_RANDOMIZED |Intervention Model: SINGLE_GROUP |Masking: NONE |Primary Purpose: TREATMENT	COMPLETED	Calithera Biosciences, Inc

## Results

### Case description

**Fig. 1:**
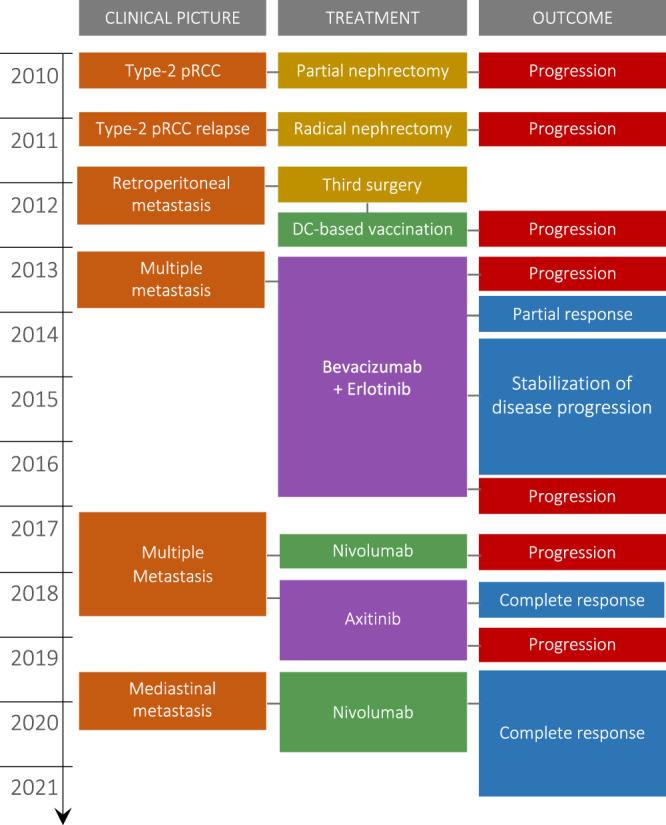
Case report timeline. Description of the clinical picture, treatment, and outcome during the patient’s 10-year follow-up.

A 46-year-old male patient with a history of hereditary (formerly type 2) papillary renal cell carcinoma (pRCC) had a tumor detected in the right kidney and underwent a partial nephrectomy in 2010, followed by relapse and radical nephrectomy in 2011 (Fig. 1). Anatomopathological analysis revealed well-defined encapsulated papillary formations with fibrovascular centers surrounded by round nuclei, slightly pleomorphism, prominent nucleoli or intranuclear inclusions, eosinophilic cytoplasm, and 3-4 mitotic figures every ten fields, with peritumoral interstitial nephritis. Immunohistochemical analysis revealed intense tumoral reactivity for vimentin, whereas CD10 and CK7 markers were negative. Ki67 determined up to 10% cellular proliferative rate in areas of major neoplastic growth. Further investigation through genomic sequencing confirmed a germline heterozygous *FH* mutation c.1349_1352delATGA at cDNA level or p.Asn450SerfsX3 at the protein level. The c.1349_1352delATGA mutation in the *FH* gene causes a frameshift starting with codon Asparagine 450, changes this amino acid to a Serine residue, and creates a premature stop codon at position 3 of the new reading frame (p.Ans450SerfsX3) resulting in protein truncation, predicted to cause loss of normal protein function^29^. As such, the ACMG guidelines would likely classify the c.1349_1352delATGA (p.Asn450SerfsX3) variant as pathogenic^30,31^. To date, this syndrome was characterized by the absence of cutaneous leiomyomatosis in the patient and the nine individuals from his family who passed due to metastatic renal cancer; the heterozygous *FH* mutation was identified in six family members (including the proband) among the twenty individuals tested during genetic counseling^29^.

**Fig. 2:**
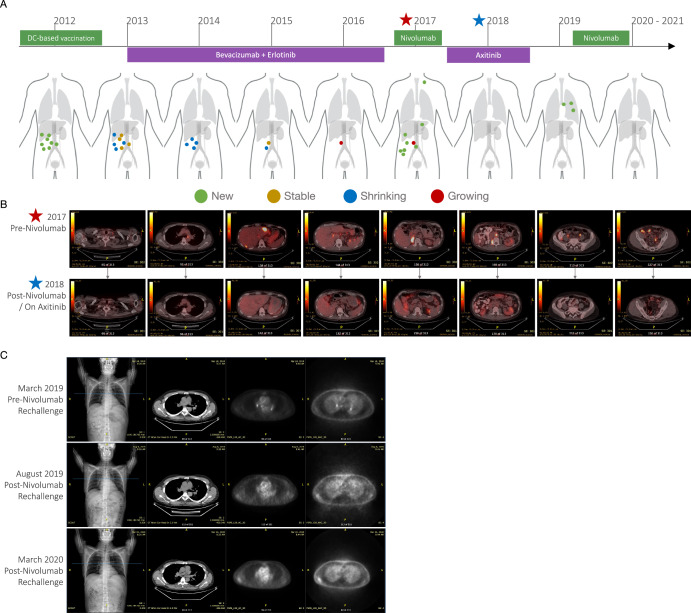
Striking complete response to nivolumab and axitinib. **A** Timeline of radiological response by RECISTv1.1 criteria during immunotherapy. **B** PET-CT scans pre and post nivolumab / during axitinib. **C** PET-CT scans pre and post nivolumab rechallenge.

**Fig. 3:**
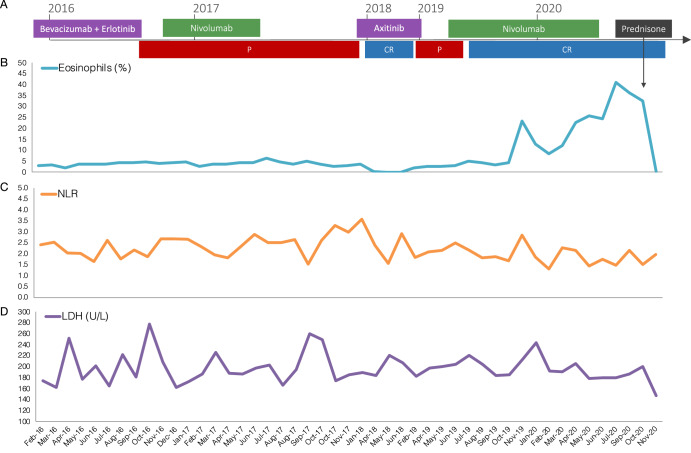
Biomarker follow-up during nivolumab. **A** Timeline of treatment and outcome during immunotherapy (P progression, CR complete response). **B** Follow-up of circulating eosinophils. **C** Follow-up of neutrophil to lymphocyte ratio (NLR). **D** Follow-up of serum levels of lactate dehydrogenase (LDH).

In 2012, a retroperitoneal metastasis was detected, and the patient underwent a third surgery and enrolled in a dendritic cell (DC)-based vaccination protocol at the University of Sao Paulo, Brazil. He received four intradermal doses of 1.5 ×10^7^ irradiated DC-tumor cell hybrid cells (healthy donor monocyte-derived DCs hybridized with patient-derived tumor cells) in 1 ml of sterile saline, with a 1-month interval between doses^32^. Although the follow-up of the patient’s immune function showed significant changes compatible with the break of immunosuppression and immune restoration^33^, three months after vaccination, five abdominal hypermetabolic nodules were observed during FDG PET evaluation (Fig. 2A). The patient was started on bevacizumab i.v. 10 mg/kg every 2 weeks and erlotinib 100 mg daily; after nine months of treatment, follow-up imaging revealed partial response by RECISTv1.1 criteria followed by stabilization of disease progression for about 45 months. By the end of 2016, imaging follow-up showed signs of progression, and by mid-2017, the patient was started on nivolumab (anti-PD-1), i.v. 3 mg/kg/dose followed by a 200 mg flat dose, due to skin toxicity. Three months later, follow-up imaging revealed progression –perhaps pseudoprogression, leading to a transient therapeutic regimen switch for axitinib 10 mg and off-label metformin 500 mg daily for a year. During this period and since mid-2018, he demonstrated complete metabolic response through NMPET analysis according to RECISTv1.1 criteria (Fig. 2B). During axitinib, new mediastinal lesions anticipating disease progression in early 2019 were completely reverted upon nivolumab regimen reestablishment (Fig. 2C). By mid-2020, nivolumab was discontinued due to sustained eosinophilia (unresolved with empiric antiparasitic treatment and after ruling out organ damage and myeloproliferative neoplasm). Eosinophilia persisted for three additional months off-nivolumab until managed with prednisone 70 mg for 10 days followed by 10 mg for a month (Fig. 3A, B). Despite undergoing several side effects, such as skin toxicity, thyroiditis, fibromyalgia, migraine, and eosinophilia (all managed by different specialties; Supplementary Table 1), he sustained the successful complete response to nivolumab as of January 2021, when the patient voluntarily withdrew any further oncological follow-up or treatment. Regardless of the many side effects experienced during the 10-year treatment, the patient’s activities of daily living, including working, were not disrupted by long-term hospitalization or disability. Nevertheless, the patient opted for assisted dying in December 2021.

### Eosinophilia and tumor expression analysis of *SIGLEC8*

**Fig. 4:**
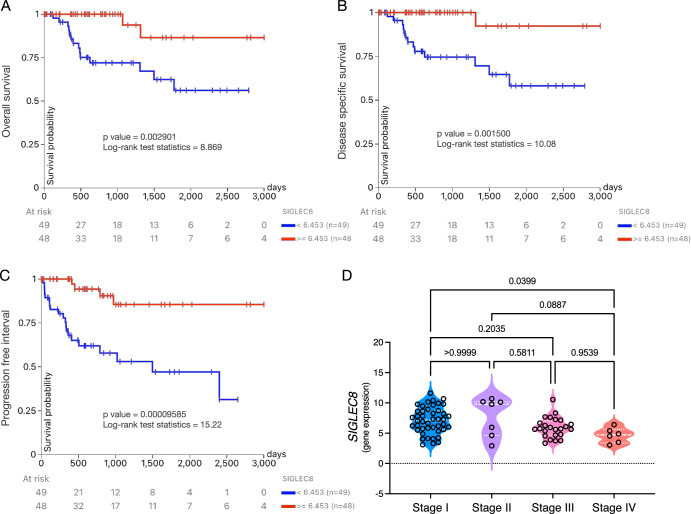
*SIGLEC8* as a biomarker of response to nivolumab in (formerly type 2) pRCC. Analysis of the TCGA Kidney Papillary Cell Carcinoma dataset using Xena^52^. Only samples from primary type 2 pRCC tumors were included, totaling 97 samples with 3000 days follow-up. The prognostic value of high (≥6.453, red) or low (<6.453, blue) *SIGLEC8* expression in tumors was assessed through its capacity to predict better or worse: **A** Overall survival, **B** Disease-specific survival, and **C** Progression-free interval. **D** *SIGLEC8* tumor expression determined by RNAseq was analyzed among type 2 pRCC pathological stages; dotted white lines refer to quartiles, and dashed white lines refer to the median. Data were analyzed using the Kruskal-Wallis test with Dunn’s multiple comparison test.

High serum lactate dehydrogenase (LDH) and neutrophil-to-lymphocyte ratio (NLR) have been associated with worse overall survival in cancer patients from different etiologies treated with immune checkpoint inhibitors (ICI), frequently used as biomarkers to predict outcomes and immune-related adverse events (irAEs)^34–36^. Here, neither showed any predictive trend before, during, or after nivolumab treatment (Fig. 3C, D). In contrast, only blood eosinophil frequency and absolute counts dramatically increased after nivolumab reestablishment and were sustained during complete response (Fig. 3B and Supplementary Fig. 1). Mechanistically, immunotherapy stimulates the production of IL-5 and IL-33 by CD4+ T cells, which induces the production of eosinophils in the bone marrow, increased systemic eosinophil circulation, and accumulation in the tumor site. Using an eosinophil gene signature (*SIGLEC8, RNASE2, RNASE3, IL5RA*, and *CCR3*), it was found that eosinophil infiltration inside the tumor correlates with increased CD8+ T cell and IFN-γ signatures and was shown that tumor eosinophils enhance CD8+ T cell activation, leading to tumor eradication^37^. As an isolated fact, during the patient’s investigation of eosinophilia, CD8+ T cells were found to predominate amongst other lymphocytes in the bone marrow aspirate. A preliminary analysis of the TCGA Kidney Papillary Cell Carcinoma (type 2) dataset showed that high *SIGLEC8* expression in tumors alone leads to increased overall survival (*p* = 0.002901, Fig. 4A), disease-specific survival (*p* = 0.001500, Fig. 4B) and progression-free interval (*p* = 0.00009585, Fig. 4C). Except for the progression-free interval using *IL5RA* (*p* = 0.01690, Supplementary Fig. 2D), the eosinophil gene signature^37^ or any of its individual genes did not present any prognostic value or expression differences among pathological stages (Supplementary Fig. 2). In contrast, tumor expression of *SIGLEC8* was significantly higher at early pathological stages compared to stage IV (Fig. 4D); combining formerly type 1 and 2 pRCC showed similar results (Supplementary Fig. 3). Certainly, *SIGLEC8* expression was shown to be increased in tumors from ICI responders compared to non-responders^37^. Eosinophilia has been identified as a marker of favorable prognosis in cancer patients treated with ICI but also predictive of toxicity and irAEs^34,38–41^. Besides eosinophilia, in this case, no other irAEs were induced by nivolumab, nor eosinophilia-related toxicity.

## Discussion

In this case of FH-deficient pRCC, a heavily pre-treated patient who responded remarkably to late nivolumab rechallenge accompanied by eosinophilia illustrates the potential of immunotherapy in managing such aggressive malignancies. When diagnosed with pRCC in 2010, the patient documented here was given months to live based on the poor prognosis of other family members. Unlike his relatives, the patient lived more than 10 years after diagnosis and for the last 2 years, he lived cancer-free. When new metastatic lesions appeared in the lungs during axitinib treatment, nivolumab rechallenge was recommended based on previous evidence of immune status restoration (break of tumor-induced anergy and tolerance) observed after DC-based vaccination^33^, the possibility of pseudoprogression after the first nivolumab regime, lower toxicity compared to ipilimumab (the other ICI option approved at the time) and availability in the patient’s home country. The rechallenge with ICI in mRCC has shown limited effectiveness^26–28^, although, in melanoma, the use of microbiome interventions (fecal microbiome transplant, FMT) has proven to overcome ICI resistance in some patients and improve response to subsequent ICI rechallenge^42,43^. In mRCC, FMT and live bacterial supplementation (CBM588) are currently being tested in the first-line setting; the role of a microbiome intervention in ICI rechallenge remains to be studied^44–46^. Here, ICI rechallenge was an uncommon but successful decision, defying recent discouragement for mRCC^28^.

The timing in which immunotherapy and other therapies were given, when a possible immune response was already in progress or tumor burden was still low, may have played an important role in the success of this case. A possible pseudoprogression phenomenon due to immunotherapy was observed here, firstly suspected after DC-based vaccination where disease progression was observed during the vaccine protocol followed by a partial response and long-term disease stabilization (tumor dormancy?) shortly after inclusion of bevacizumab plus erlotinib regimen. A second and more apparent pseudoprogression was observed when nivolumab was first introduced, leading to its premature discontinuation. Axitinib’s role in pseudoprogression and during the initial disease control could not be ruled out, nor its possible synergistic effect with metformin^2,47^. Unfortunately, the disease progressed to a new organ while on axitinib treatment. The re-establishment of nivolumab clearly showed how this patient was, in fact, a responder beyond disease progression per RECISTv1.1, supporting the idea that patients like him would benefit from treatment rechallenge. For about two decades, ICI has been used in the clinical setting, however, pseudoprogression still represents a challenge due to a lack of awareness and ICI-specific radiological criteria. There is an urgent need to accurately differentiate pseudoprogression (due to inflammation, immune cell infiltration, edema, or necrosis) from actual progression^48^, in a manner that can be easily implemented in any clinical setting that provides immunotherapy.

From the cancer immunology perspective, tumor dormancy occurs when the immune system establishes equilibrium with the tumor after an antitumor immune response. This equilibrium is broken when the antitumor effector immune cells naturally die, leading to less immune pressure over the remaining tumor cells (quiescent tumor cells, cancer stem cells) and tumor escape^49^. Tumor dormancy might have been induced here by the DC-based vaccination and sustained by years on bevacizumab plus erlotinib; eventually, the antitumor effector cells die without engaging in further clonal expansion due to the lack of tumor antigens. Patients in situations like this would likely benefit from targeting tumor dormancy with further immunotherapy strategies such as cytokines (IL-15) that reinvigorate antitumor effector cells such as NK cells and CD8^+^ T cells^50,51^. Understanding the timing of a patient’s tumor and its microenvironment from the cancer immunology perspective will be crucial for the effective recommendation of different immunotherapy modalities in the future.

This case highlights the potential of ICIs, particularly ICI rechallenge as a viable option for treating heavily pre-treated patients with aggressive forms of RCC such as *FH*-deficient. Moreover, underlines the presence of eosinophilia during ICI treatment as a favorable indicator of immune activation, heralding a strong antitumor response. The growing evidence from case reports emphasizes the potential of immunotherapy^21–24^, perhaps in combination with TKIs^25^, as an effective strategy for managing this challenging malignancy. Cases like this exemplify how immunotherapy is turning cancer into a chronic disease; still, more needs to be done to decrease the risk of long-term adverse events that significantly deteriorate the quality of life of cancer-free patients.

## Methods

Please see above for details of the patient’s presentation and clinical history. The patient involved in this study and his family provided the authors with written informed consent for publication, available to the editors upon request. The follow-up of this case was performed in agreement with the principles embodied in the Declaration of Helsinki and was approved by the Research Ethics Committee of the institution where the follow-up started, where M.A.C-S. was affiliated from 2010 until 2020 (Comissão de Ética em Pesquisas com Seres Humanos, No. 969/CEP, Institute of Biomedical Sciences, University of Sao Paulo, Brazil).

### TCGA analysis

The analysis of the TCGA Kidney Papillary Cell Carcinoma dataset was done using Xena^52^. *SIGLEC8* expression in tumors was assessed through its capacity to predict better or worse overall survival, disease-specific survival, and progression-free interval. Samples analyzed were from either primary pRCC tumors (totaling 287 samples with 3000 days follow-up, with a cut-off for *SIGLEC8* expression in tumors of high ≥ 6.240 or low < 6.240) or only from primary type 2 pRCC tumors (totaling 97 samples with 3000 days follow-up, with a cut-off for *SIGLEC8* expression in tumors of high ≥ 6.453 or low < 6.453). *SIGLEC8* tumor expression determined by RNAseq was analyzed among type 2 pRCC pathological stages. Data were analyzed in Prism using the Kruskal-Wallis test with Dunn’s multiple comparison test.

## Supplementary information

Supplementary Data

Supplementary Data 1

Supplementary Data 2

Supplementary Data 3

Supplementary Data 4

## Data Availability

The datasets used during the current study are available from the corresponding author upon reasonable request or included as Supplementary Data.
