# Supplementary material for: Complete response to fifth-line anti-PD-1 rechallenge in fumarate hydratase-mutated papillary renal cell carcinoma
Source: NPJ Precis Oncol. 2024 Nov 4;8:251. doi: 10.1038/s41698-024-00750-3 (PMC11535469; doi:10.1038/s41698-024-00750-3)
Supplement: Supplementary file 1 — Supplementary Data [file 41698_2024_750_MOESM1_ESM.pdf]

Supplementary Table 1. Side effects experienced during nivolumab rechallenge.

AE/irAE	Specialty	Severity	Clinical management	Status
Skin toxicity	Dermatology	Moderate	Minocycline 200 mg PO daily	Managed
Thyroiditis	Endocrinology	Moderate	Levothyroxine 50 mcg PO daily	Managed
Fibromyalgia* / Symptoms included asthenia, generalized pain, insomnia paresthesia, hyperesthesia and nausea.	Internal Medicine, Rheumatology, Pain management and Palliative care.	Severe	Tapentadol, 50 mg PO twice a day, increased up to 500 mg daily if needed. Switched to Pregabalin 150 mg PO daily and Duloxetine 30 mg PO daily.	Managed but unsolved
Migraine	Neurology	Severe	Sumatriptan, 100 mg single dose. Switched to Olanzapine, 5 mg PO daily	Managed
Eosinophilia**	Internal medicine and Hematology	Moderate	Prednisone 70 mg PO daily for 10 days followed by 10 mg PO daily for a month.	Resolved

*Normal CPK.

**Empirically treated with albendazole 400 mg, PO single dose. Several tests for myeloproliferative neoplasia were done, all negative. Several tests to rule out autoimmune disorders and organ damage were done, all negative.

Supplementary Figure 1

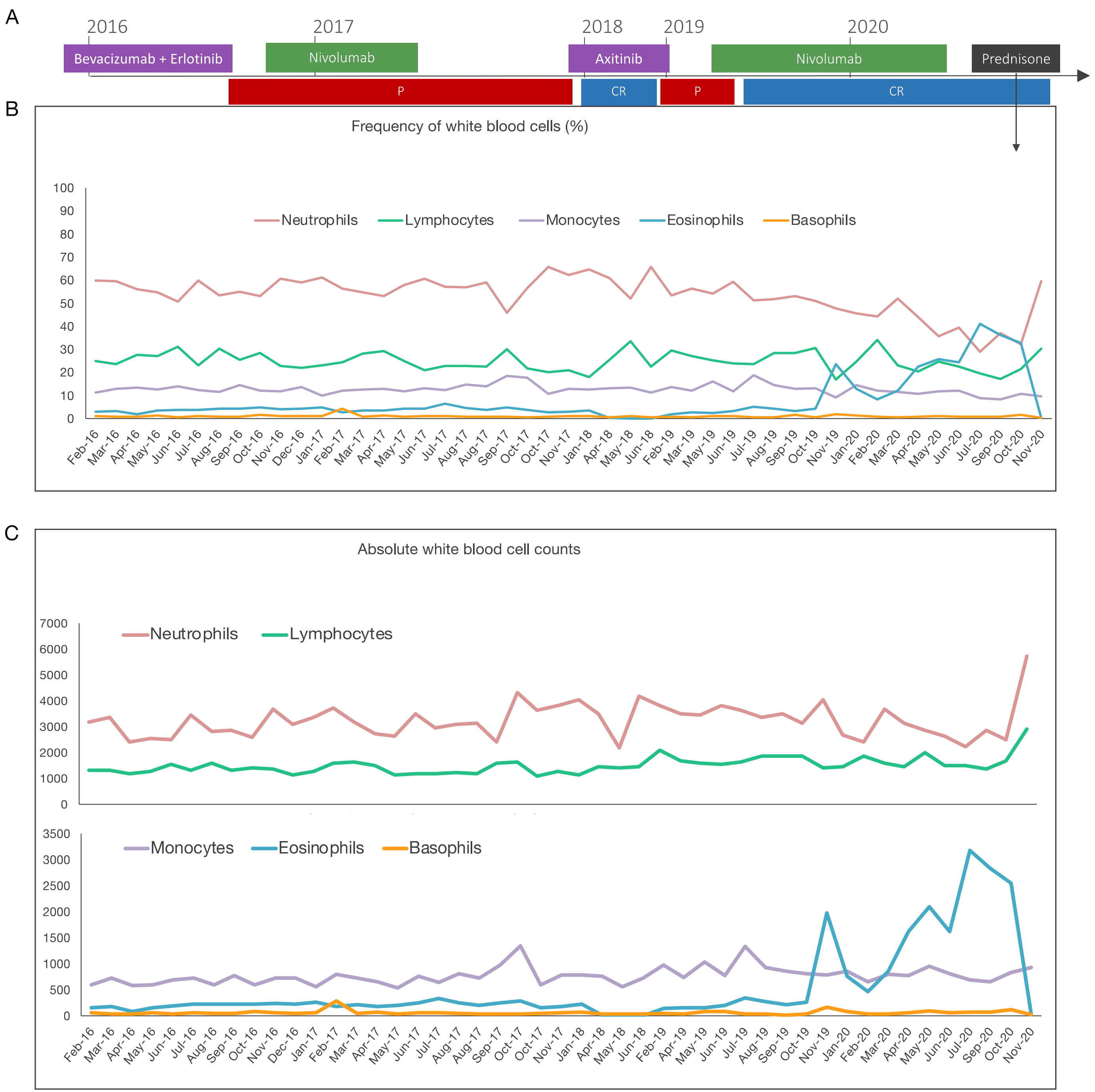

Supplementary Figure 1. A) Timeline of treatment and outcome during immunotherapy (P = progression; CR = complete response). B) Follow-up of circulating white blood cells: B) frequency and C) absolute counts.

Supplementary Figure 2

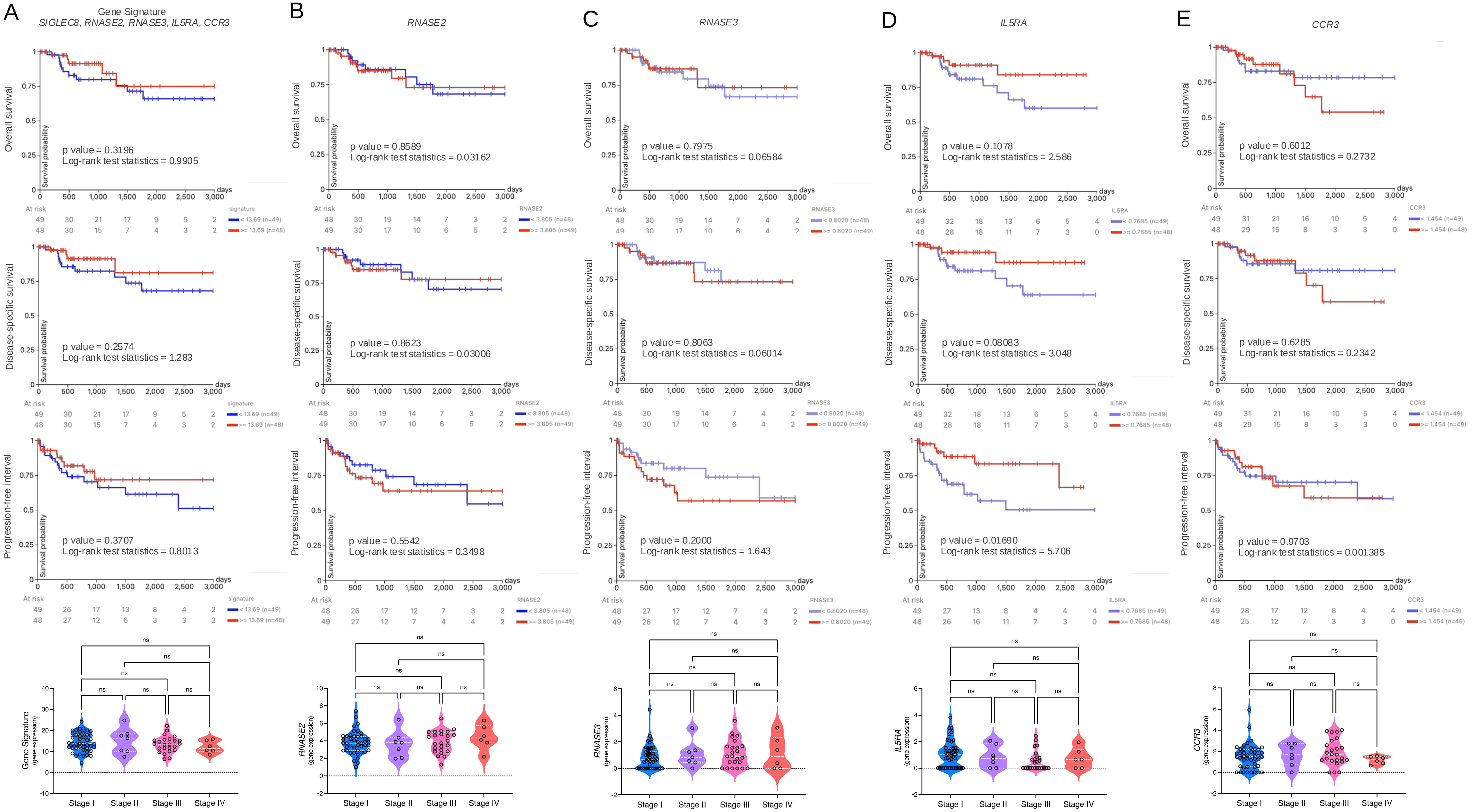

Supplementary Figure 2. Analysis of the TCGA Kidney Papillary Cell Carcinoma dataset using Xena [52]. Only samples from primary type 2 pRCC tumors were included, totaling 97 samples with 3000 days follow-up. The prognostic value of high (red) or low (blue) expression in tumors of, A) eosinophil gene signature (*SIGLEC8, RNASE2, RNASE3, IL5RA*, and *CCR3)*, B) *RNASE2*, C) *RNASE3*, D) *IL5RA*, and E) *CCR3* were assessed through its capacity to predict better or worse: Overall survival, Disease-specific survival, and Progression-free interval. The tumor expression of the eosinophil gene signature (*SIGLEC8, RNASE2, RNASE3, IL5RA*, and *CCR3)*, and each individual gene *RNASE2*, *RNASE3*, *IL5RA*, and *CCR3* determined by RNAseq was analyzed among type 2 pRCC pathological stages; dotted white lines refer to quartiles, and dashed white lines refer to the median. Data was analyzed using the Kruskal-Wallis test with Dunn's multiple comparison test.

Supplementary Figure 3

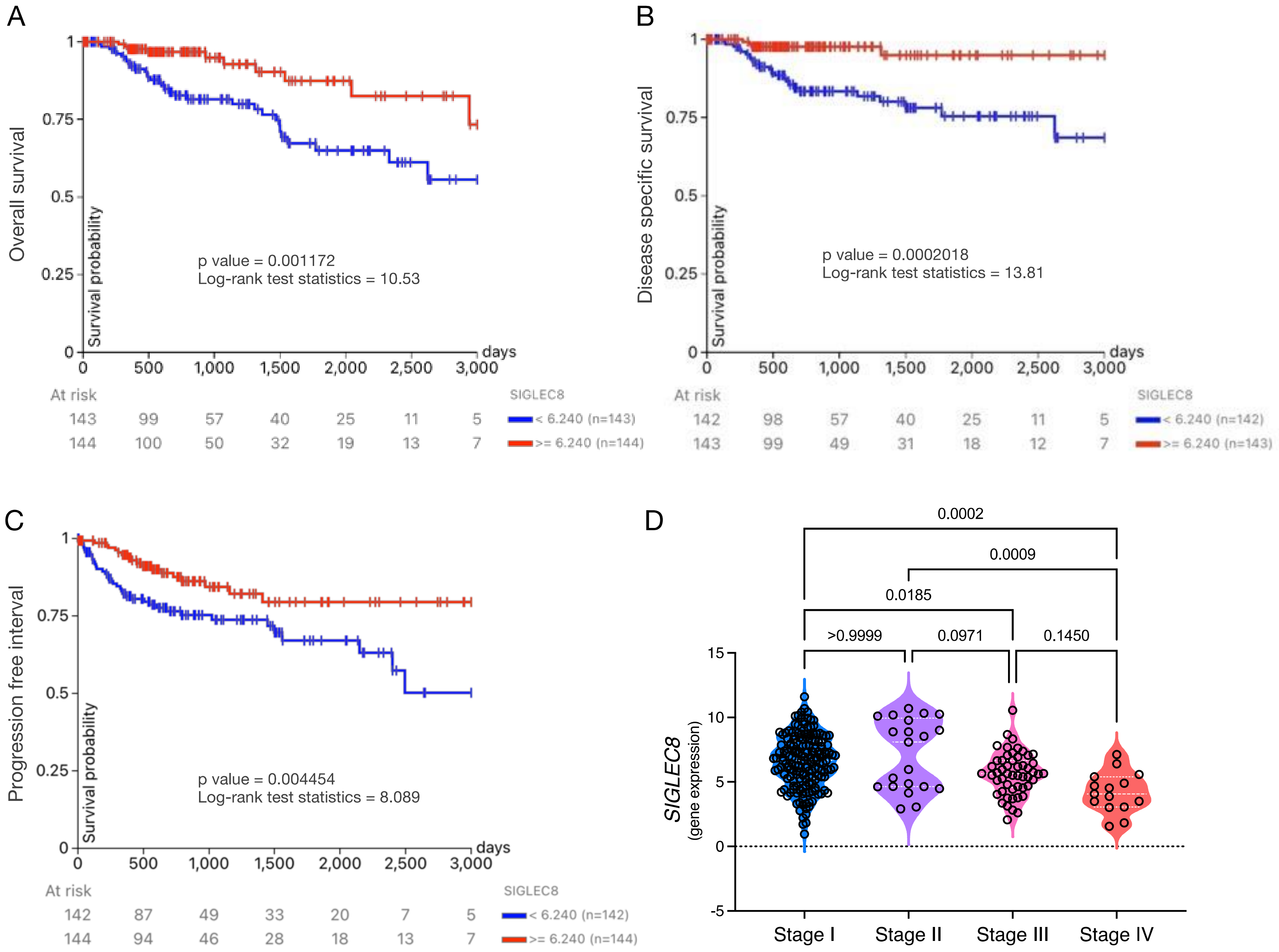

Supplementary Figure 3. *SIGLEC8* as a biomarker of response to Nivolumab in (formerly type 1 and 2 combined) pRCC. Analysis of the TCGA Kidney Papillary Cell Carcinoma dataset using Xena [52]. Samples from primary pRCC tumors were included, totaling 287 samples with 3000 days follow-up. The prognostic value of high (> = 6.240, red) or low (< 6.240, blue) *SIGLEC8* expression in tumors was assessed through its capacity to predict better or worse: A) Overall survival, B) Disease-specific survival, and C) Progression-free interval. D) *SIGLEC8* tumor expression determined by RNAseq was analyzed among pRCC pathological stages; dotted white lines refer to quartiles, and dashed white lines refer to the median. Data was analyzed using the Kruskal-Wallis test with Dunn’s multiple comparison test.
